# Optimizing esthetic zone periodontal regeneration in a 1–2‐wall infrabony defect using recombinant human platelet‐derived growth factor BB and β‐tricalcium phosphate: A case report

**DOI:** 10.1002/cre2.908

**Published:** 2024-05-26

**Authors:** Kantapon Rattanaprukskul, Rodrigo Neiva, Jonathan Korostoff

**Affiliations:** ^1^ Department of Periodontics, School of Dental Medicine University of Pennsylvania Philadelphia Pennsylvania USA; ^2^ Department of Periodontology, Faculty of Dentistry Chulalongkorn University Bangkok Thailand

**Keywords:** biologic, case report, periodontal regeneration, platelet‐derived growth factor

## Abstract

**Objective:**

Periodontitis is an inflammatory condition induced by subgingival bacterial dysbiosis, resulting in inflammatory‐mediated destruction of tooth‐supporting structures, potentially leading to the formation of infrabony defects. This case report describes the treatment of a patient who presented with a combination 1–2‐wall defect on tooth 21. To maintain the residual periodontal attachment and minimize esthetic consequences, a regenerative approach was performed using recombinant human platelet‐derived growth factor‐BB (rh‐PDGF‐BB) and β‐tricalcium phosphate (β‐TCP).

**Materials and Methods:**

At the time of postscaling/root planing reevaluation, a 34‐year‐old Asian male initially diagnosed with molar/incisor pattern stage III grade C periodontitis exhibited a 6‐mm residual probing depth on the mesiopalatal aspect of tooth 21. Periodontal regenerative surgery was performed using rh‐PDGF‐BB with β‐TCP, without the use of a membrane.

**Results:**

At the 1‐year follow‐up, a significant reduction in probing depth and radiographic evidence of bone fill were observed. Additionally, re‐entry surgery for implant placement at site tooth 23 confirmed bone fill in the defect on tooth 21.

**Conclusion:**

These results demonstrate the efficacy of rh‐PDGF‐BB with β‐TCP in enhancing periodontal regeneration and support its use as a treatment option when treating poorly contained infrabony defects in the esthetic zone.

## 1 INTRODUCTION

Periodontitis is a chronic inflammatory disease triggered by deregulated inflammation induced by subgingival microbial dysbiosis, resulting in the destruction of the tooth‐supporting structures: cementum, alveolar bone, and periodontal ligament (PDL) (Hajishengallis, 2015; Hajishengallis et al., 2012). The Center for Disease Control reports that almost one‐half of the adult population in the United States exhibits periodontitis (Eke et al., 2015). Although the condition has been recognized as a disease entity for centuries, it continues to be a major health burden worldwide (Kassebaum et al., 2014). The goal of periodontal therapy is to eliminate the pathogenic microflora and restore the injured periodontal tissue to a state of health by either a regenerative or reparative form of wound healing. Guided tissue regeneration (GTR) is the gold standard for periodontal regenerative surgery. The central concept of GTR is to utilize a barrier membrane that physically excludes soft tissue cells from migrating into a periodontal defect and allows cells with regenerative potential “selective” access to the site. It is well known that epithelial downgrowth into a healing defect interferes with regenerative processes and leads to the less desirable outcome of repair via a long junctional epithelial attachment (Position Paper, 2005). The efficacy of GTR has been well‐documented for the regeneration of infrabony and furcation defects (Kao et al., 2015; Position Paper, 2005).

The term “biologic” refers to a class of drugs derived from biological sources or manufactured via recombinant DNA technology that are utilized to treat patients suffering from a wide array of diseases, including periodontitis. Although the mechanisms of action of the compounds vary, molecules have been identified that promote periodontal regeneration, including platelet‐derived growth factor (PDGF), enamel matrix derivative (EMD), and fibroblast growth factor (FGF) (Avila‐Ortiz et al., 2022). PDGF is a polypeptide growth factor that regulates cell proliferation and facilitates wound healing. In 1989, Lynch and colleagues demonstrated that PDGF facilitates periodontal regeneration in canines (Lynch et al., 1989). Subsequent studies also showed that recombinant human PDGF subtype BB (rh‐PDGF‐BB) is the most potent isoform of PDGF relative to periodontal regenerative activity (Darby & Morris, 2013; Tavelli et al., 2021). Currently, rh‐PDGF‐BB is sold under the trade name GEM‐21. The recombinant protein is intended for use along with a carrier, beta‐tricalcium phosphate (β‐TCP), which is a multicrystalline and porous form of calcium phosphate mimicking the mineral component of natural bone. β‐TCP is osteoconductive and has been approved by the Food and Drug Administration (FDA) as a synthetic bone graft material for use in humans. Studies involving the treatment of infrabony defects in human subjects showed that the combination of rh‐PDGF‐BB and β‐TCP yielded exceptional clinical outcomes including significant gain in attachment and minimal gingival recession (Nevins et al., 2005). Furthermore, the clinical improvements exhibited long‐term stability (Avila‐Ortiz et al., 2022). Because of the porous structure of β‐TCP, it absorbs rh‐PDGF‐BB and releases it slowly to facilitate the regenerative process. Recently, the American Academy of Periodontology best evidence consensus statement on the use of biologics in clinical practice acknowledged the efficacy of using rh‐PDGF‐BB to treat infrabony defects (Avila‐Ortiz et al., 2022).

In spite of improvements in surgical techniques and the availability of novel materials, the treatment of 1–2‐wall and noncontainable infrabony defects remains a clinical challenge. Additionally, the utilization of barrier membranes may lead to the undesirable consequence of gingival recession, a concern of particular significance when the treatment site lies within the esthetic zone (Ling et al., 2003). In this case report, we described the treatment of a patient presenting with a 1–2‐wall bony defect on the maxillary left central incisor (tooth 21). Recognizing the potential for a membrane to cause recession and the ability of rh‐PDGF‐BB to facilitate periodontal regeneration, we opted to treat the patient using GEM‐21 without a membrane, as per the manufacturer's protocol (Avila‐Ortiz et al., 2022; Ling et al., 2003). Furthermore, a re‐entry procedure was performed at the surgical site, demonstrating substantial bone regeneration within the defect. Taken together, the clinical findings suggest that this approach results in periodontal regeneration without compromising the esthetic outcome.

## 2 MATERIALS AND METHODS

A 34‐year‐old Asian male presented to the Post‐Graduate Periodontics/Periodontal Prosthesis Clinic at the University of Pennsylvania School of Dental Medicine (PDM) with a chief complaint of “I have severe gum disease.” The patient was classified as ASA II with a history of recreational drug usage. The patient smoked cannabis every day for 20 years up until he started to undergo care at PDM. Periodontal examination revealed 8 mm of probing depth on the mesial and palatal aspects of the maxillary left central incisor (tooth 21) in the presence of bleeding on probing (BOP) and suppuration. Probing depths of 5–7 mm were detected on the maxillary right first molar (tooth 16), and maxillary left first molar (tooth 26) with BOP. Radiographic evaluation revealed vertical infrabony defects on the maxillary right first molar, maxillary left central incisor, and maxillary left first molar (teeth 16, 21, and 26). The patient was diagnosed with molar/incisor pattern stage III grade C periodontitis. The patient was advised to undergo comprehensive periodontal therapy, followed by orthodontic treatment. He was amenable to the proposed periodontal treatment plan but was not interested in orthodontic treatment. The patient provided informed consent for all dental procedures as well as the use of clinical data and images for academic purposes including publications.

**Figure 1:**
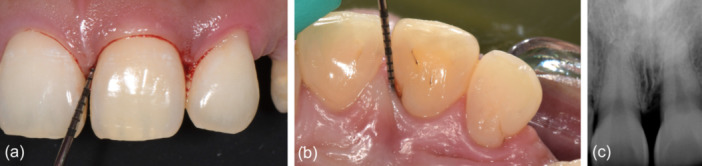
Preoperative clinical presentation of the maxillary left central incisor (tooth 21). (a) Facial view. (b) Palatal view demonstrating 6 mm mesiopalatal probing depth. (c) Radiographic image showing an infrabony defect on the mesial aspect of the tooth.

Phase I therapy included oral hygiene instruction, occlusal adjustment, and scaling/root planing (SRP). The SRP was completed under local anesthesia (2% lidocaine with 1:100,000 epinephrine) in conjunction with a 7‐day course of antibiotic treatment (500 mg amoxicillin + 250 mg metronidazole TID). Six weeks after SRP, periodontal reevaluation was done revealing a residual 6 mm probing depth on the mesiopalatal aspect of the maxillary left central incisor (tooth 21) (Position Paper, 2004; Segelnick & Weinberg, 2006) (Figure 1a–c). Periodontal regenerative surgery was recommended and accepted by the patient. A written informed consent was signed by the patient.

**Figure 2:**
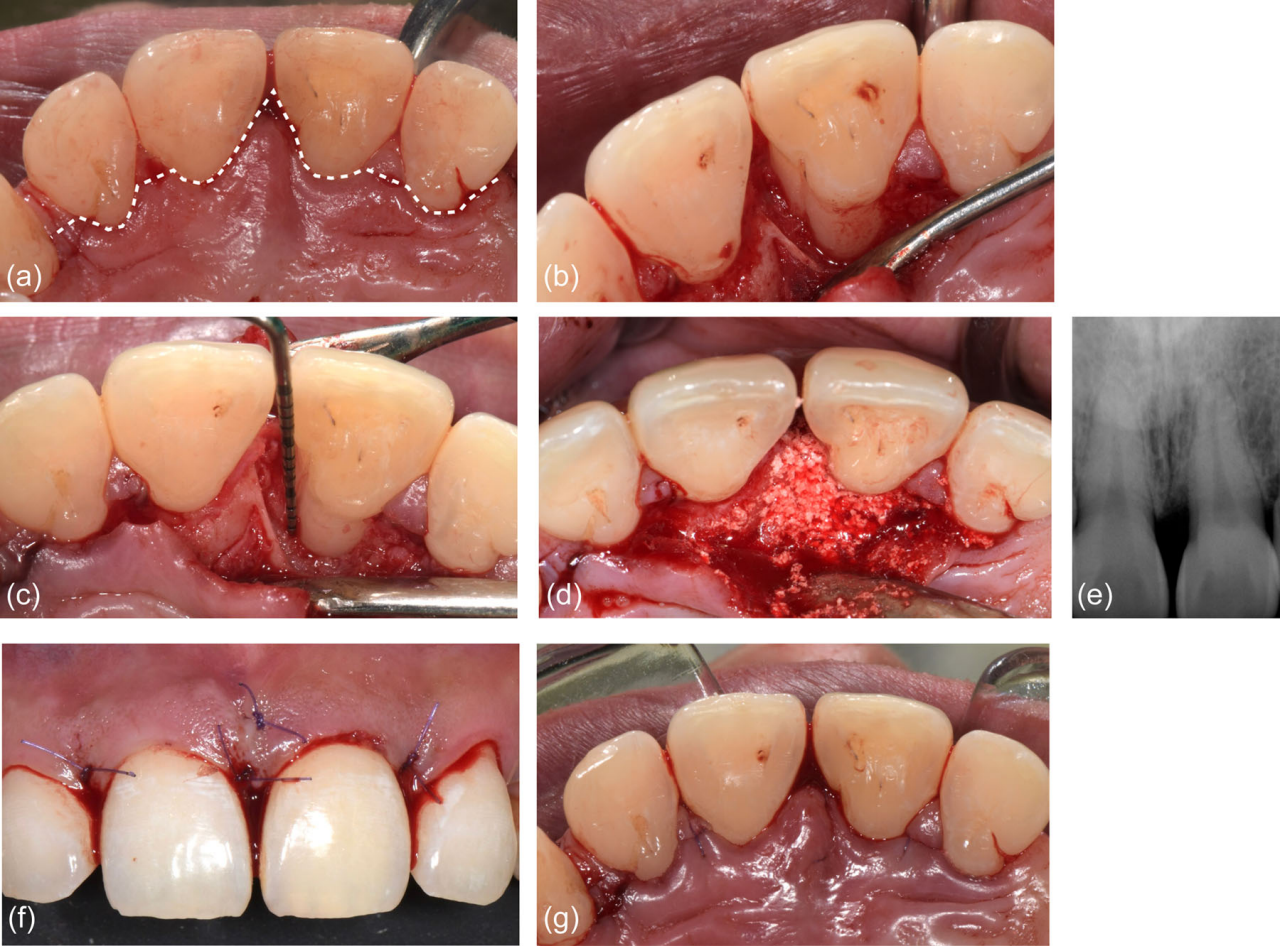
Regenerative periodontal surgical procedure. (a) A sulcular palatal incision with interproximal papilla preservation was made from the maxillary right lateral incisor to the maxillary left lateral incisor (teeth 12–22) and a full thickness mucoperiosteal flap on the palatal was elevated. (b) 1–2‐wall infrabony defect with palatogingival groove of the maxillary left central incisor (tooth 21). (c) 1–2‐wall 8 mm deep infrabony defect on the mesiopalatal aspect of maxillary left central incisor after odontoloplasty of palatogingival groove. (d) Application of β‐TCP pretreated with rh‐PDGF‐BB solution (GEM‐21) over the bony defect. (e) Radiographic image following graft application demonstrating complete fill of the defect with the radiopaque material. (f and g) Use of 5‐0 PGA‐PCL copolymer (Glycolon) to achieve tension‐free primary closure of the facial and palatal flaps, respectively.

Following administration of local anesthesia (2% lidocaine with 1:100,000 epinephrine), a sulcular palatal incision with interproximal papilla preservation was made from maxillary right lateral incisor to maxillary left lateral incisor (teeth 12–22), adhering to the principles of the single‐flap approach, which aims to reduce surgical trauma (Figure 2a) (Trombelli et al., 2009). A full‐thickness mucoperiosteal palatal flap was meticulously elevated. Granulation tissue was removed from between the teeth revealing a deep 1–2 wall infrabony defect encompassing the mesial and palatal aspects of the maxillary left central incisor (tooth 21). Additionally, a palatal groove was identified and subsequently eliminated through odontoplasty, utilizing high‐speed rotary instrumentation with copious sterile saline (Figure 2b,c).

After hydration of the β‐TCP with the rh‐PDGF‐BB solution (GEM‐21) for 10 min, the material was placed and firmly adapted to the defect (Figure 2d,e). Tension‐free primary closure of the flaps was achieved via a combination of horizontal mattress and simple interrupted 5‐0 polyglycolic acid‐polycaprolactone copolymer sutures (Glycolon) (Figure 2f,g). The patient was provided with comprehensive postoperative instructions, encompassing a prescription for 500 mg of amoxicillin to be taken every 8 h over a period of 7 days, along with 600 mg of ibuprofen available for use every 4–6 h as required for pain management. With regard to oral hygiene, the patient was instructed to refrain from brushing the surgical site for a duration of 2 weeks, while emphasizing the importance of rinsing with warm salt water twice daily. The patient also was advised to avoid consuming hot or acidic foods and beverages.

The patient returned for a follow‐up visit 2 weeks after the surgery and reported no pain or discomfort. The healing of the surgical site was found to be uneventful. The sutures were removed, and the surgical site was gently deplaqued with hand instruments. Additional follow‐up visits were scheduled at 4 weeks, 2 months, and 3 months postsurgery to evaluate the progression of healing. The patient was placed on a 3‐month maintenance recall regimen.

## 3 RESULTS

**Figure 3:**
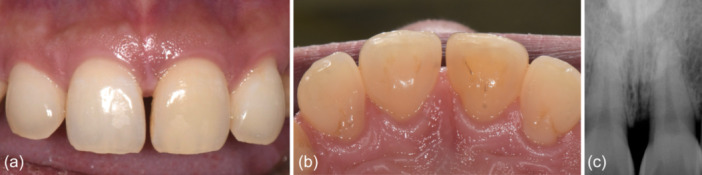
Six‐month follow‐up postsurgical follow‐up. (a and b) Excellent healing without exposure of the graft or defect on the facial and palatal, respectively. (c) Radiographic image of the maxillary left central incisor (tooth 21) showing a bone‐like radiopacity in the defect.

**Figure 4:**
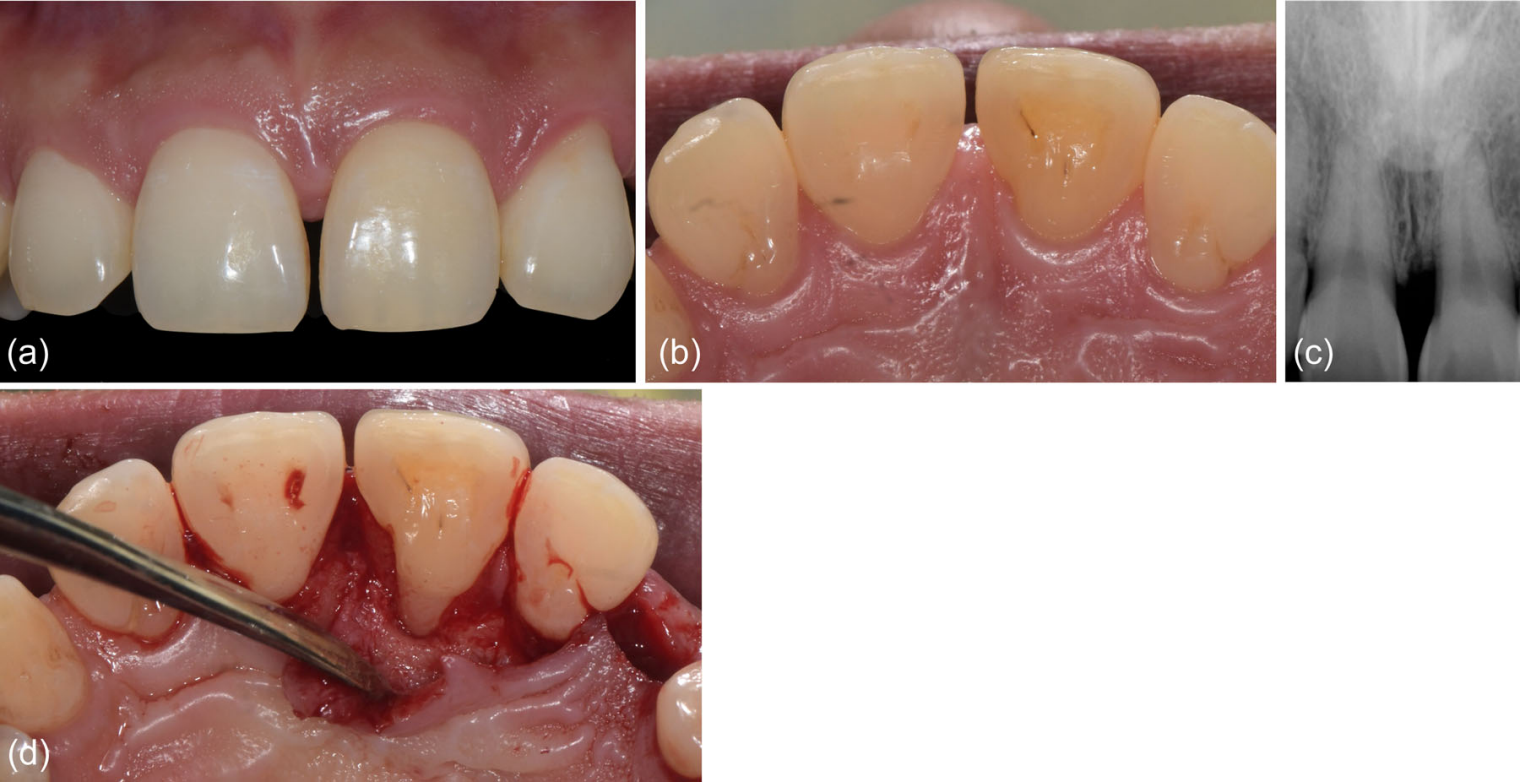
One‐year surgical re‐entry. (a and b) Excellent soft tissue healing on the facial and palatal aspects of the maxillary left central incisor (tooth 21), respectively. (c) Radiographic image showing bone‐like radiopacity in the treated defect on the mesial of the maxillary left central incisor (tooth 21). (d) Palatal view showing apparent bone fill of the defect.

**Figure 5:**
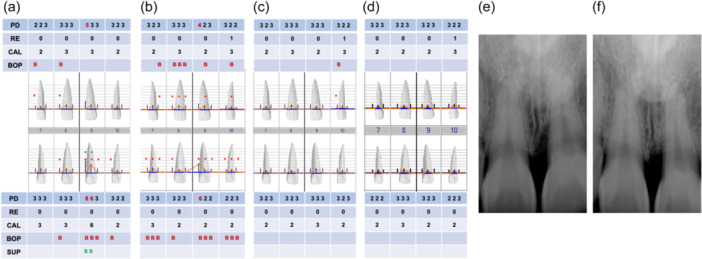
Periodontal charting and radiographic images. (a) Baseline charting before phase I therapy. (b) Six‐weeks after phase I therapy charting. (c) Six‐months postsurgical charting. (d) One‐year postsurgical charting. (e) Initial radiograph. (f) One‐year postsurgical radiograph.

The patient was seen 6 months after the surgical procedure for reevaluation charting and periapical radiographs (Figure 3a–c). Probing depths were reduced to 2–3 mm in the absence of BOP, and radiographic bone filled was observed. The 3 mm gain in clinical attachment occurred in the absence of gingival recession, thereby maintaining the esthetic appearance of the patient's smile. A year subsequent to the regenerative procedure, the patient presented for implant placement at the site maxillary left canine (tooth 23), affording an opportunity for in situ assessment of the treated defect on the maxillary left central incisor (tooth 21). There was observable evidence of 2–3 mm bone fill within the previously afflicted defect (Figures 4 and 5).

## 4 DISCUSSION

This case report demonstrates the efficacy of using the combination of rh‐PDGF‐BB and β‐TCP to successfully manage a 1–2‐wall infrabony defect on an anterior tooth. Six‐month postoperatively, the clinical assessment revealed excellent soft tissue health, marked by a substantial reduction in probing depth from 6 to 3 mm and a 3 mm gain in clinical attachment. Based on the periapical radiograph, it was suggested that the defect had undergone significant bone fill. This observation was substantiated 1 year subsequent to the procedure when we had the opportunity to surgically evaluate the site. We do believe that the previously lost attachment on the tooth was successfully regenerated.

The classic technique of GTR as initially explicated by Karring et al. involves the strategic implementation of a designated “barrier” membrane to exclude soft tissue cells from the defect to allow preferential repopulation of the site with progenitor cells capable of differentiating into cementoblasts, osteoblasts, and periodontal ligament (PDL) fibroblasts (Karring et al., 1980). Subsequently, clinicians began using bone substitute grafts in conjunction with membranes to enhance the maintenance of space for regeneration and serve as a scaffold for bone deposition. Most recently, biologics have become available that are capable of inducing periodontal regeneration when used independently or facilitating the process when used in conjunction with membranes and/or grafts. Biologics currently in use include Enamel Matrix Derivative (EMD), platelet‐rich plasma (PRP), platelet‐rich fibrin, and rh‐PDGF (Position Paper, 2005). Amongst these, clinical trials indicate that 0.3 mg/mL of rh‐PDGF‐BB used in conjunction with β‐TCP as a carrier with or without membranes can be used to predictably regenerate well‐contained osseous defects (Nevins et al., 2005). Histologic evaluation of human tissue has revealed normally oriented PDL collagen fibers inserted into new cementum and new bone formation in defects treated with rh‐PDGF‐BB (Nevins et al., 2003; Stavropoulos et al., 2010). Moreover, minimal long junction epithelial downgrowth into the defect was observed when rh‐PDGF‐BB and bone grafts were utilized without membranes (Nevins et al., 2003; Stavropoulos et al., 2010).

Per the manufacturer's instructions, rh‐PDGF‐BB is intended to be used in conjunction with β‐TCP, a highly biocompatible molecule, as its carrier. Over the years, numerous clinicians have reported on the use of a variety of different graft materials in place of β‐TCP with variable regenerative outcomes. In contrast to allografts, β‐TCP manifests a protracted resorption process. In preclinical studies, bone defects treated with β‐TCP demonstrated a significant new bone deposition at the 3‐month postsurgical milestone, nearly complete osseous reconstitution at 6 months, and ultimately achieving full resorption at 24 months. Intriguingly, no notable disparities were observed between treated sites, whether or not they were covered with a membrane (Artzi et al., 2004).

The management of the defect described in this report exhibited two challenges: its morphology and location. A major determining factor relative to predicting the success of periodontal regenerative surgery is the morphology of the osseous defect. The ideal situation is a containable defect surrounded by intact walls of bone, commonly referred to as 3‐ or 4‐wall defects, each with a depth exceeding 4 mm (Cortellini et al., 1993; Kao et al., 2015; Laurell & Gottlow, 1998; Levine et al., 2023). The defect treated for this case report would be considered as exhibiting limited regenerative potential, predominantly due to its noncontainable 1–2‐wall morphology. Regarding the location of the lesion in the esthetic zone, prudent consideration was given to the heightened susceptibility to gingival recession that may arise from the use of a barrier membrane. We implemented a single‐flap approach, strategically preserving the integrity of both the buccal gingiva and interdental papilla, thereby minimizing the surgical trauma typically associated with flap elevation (Trombelli et al., 2009). It facilitates optimal accessibility for performing the procedure with minimal postoperative recession, especially in areas of high esthetic expectations. However, it is important to acknowledge that the use of a membrane introduces the potential risk of membrane exposure and wound dehiscence, factors that could potentially compromise the ultimate success of the surgical intervention. Considering β‐TCP's capacity to serve as a scaffold for cell migration and its gradual release of rhPDGF‐BB to foster cell proliferation, all without necessitating a barrier membrane, we deemed this approach the most judicious course of action for orchestrating the regeneration of the patient's defect, all the while safeguarding against the occurrence of gingival recession.

This case report substantiates the empirical efficacy of the application of rh‐PDGF‐BB in conjunction with β‐TCP for periodontal regenerative procedures. Notably, our study stands out for its comprehensive evidence, which includes outcomes from re‐entry surgery, as well as clinical and radiographic data. Additionally, we highlight a unique aspect of regenerative surgery: the utilization of minimally invasive techniques (specifically, the single‐flap approach) with biologics, omitting the need for a membrane. Moreover, our findings underscore the achievement of surgical outcomes in 1–2‐wall defect, particularly in the anterior region, over a 1‐year follow‐up period, without compromising esthetics. However, a limitation of this study is the absence of histological analysis to illustrate the regenerated tissue. Further randomized controlled trials encompassing larger sample sizes are requisite to definitively evaluate its comparative effectiveness vis‐à‐vis alternative biologic or grafting materials.

## 5 CONCLUSION

In summary, this case report substantiates the effectiveness of utilizing rh‐PDGF‐BB and β‐TCP in the absence of a barrier membrane for the regeneration of a 1–2‐wall infrabony defect in the esthetic zone, all the while mitigating the occurrence of gingival recession. Consequently, this combination approach merits consideration in the strategic repertoire when managing an infrabony defect on a tooth within the esthetic region.

## AUTHOR CONTRIBUTIONS

Kantapon Rattanaprukskul performed the surgical procedure and follow‐up visits under the supervision of Jonathan Korostoff and Rodrigo Neiva. All three authors contributed to the preparation of the manuscript.

## CONFLICT OF INTEREST STATEMENT

The authors declare no conflict of interest.

## ETHICS STATEMENT

Written informed consent was obtained from the patient for all dental procedures as well as the use of clinical data and images for academic purposes including publications.

## Data Availability

The data that support the findings of this study are available on request from the corresponding author.
